# Prevalence and correlates of alcohol use, mental disorders, and awareness and utilization of support services among healthcare professionals in West Rand District, Gauteng, South Africa: a cross-sectional study

**DOI:** 10.1093/fampra/cmad094

**Published:** 2023-09-15

**Authors:** Charlotte Mc Magh, Oluwafojimi Fadahun, Joel Msafiri Francis

**Affiliations:** Department of Family Medicine and Primary Care, School of Clinical Medicine, Faculty of Health Sciences, University of the Witwatersrand, Johannesburg, South Africa; Department of Family Medicine and Primary Care, School of Clinical Medicine, Faculty of Health Sciences, University of the Witwatersrand, Johannesburg, South Africa; Department of Family Medicine and Primary Care, School of Clinical Medicine, Faculty of Health Sciences, University of the Witwatersrand, Johannesburg, South Africa

**Keywords:** anxiety, healthcare professionals, risky drinking, South Africa, suicide symptoms

## Abstract

**Introduction:**

Healthcare professionals (workers) are at an increased risk for developing mental and alcohol use disorders (risky drinking) due to increased psychological distress, long working hours, medical litigation, role conflict, and verbal/physical violence from colleagues and patients. Psychological well-being in healthcare workers is crucial to provide the best quality of care to patients. Current data are limited regarding alcohol abuse (risky drinking) rates and mental health condition among healthcare professionals in South Africa.

**Objectives:**

To describe the prevalence and correlates of alcohol use disorder (risky drinking), depression, anxiety, suicidality, and covid anxiety during the coronavirus pandemic in healthcare professionals in West Rand District, Johannesburg, South Africa.

**Methods:**

We carried out a cross-sectional study on a sample of healthcare professionals including doctors, nurses, clinical associates, and dentists working in the West Rand District of Gauteng, South Africa, during Covid-19 pandemic. Participants were invited to complete a paper-based questionnaire addressing sociodemographic questions, a set of measures for alcohol use disorder (AUDIT-C), depression (PHQ-2), anxiety (GAD-7), suicidality (PSS-3), covid anxiety (CAS), and awareness and utilization of support services.

**Results:**

A total of 330 healthcare professionals (60.9% nurses, 33% doctors, 5.5% other) participated. Females comprised the majority of study participants with 78.8%, and 48.2% of the participants were in the age band 35–64 years. Overall, 20.9% of the healthcare professionals reported risky alcohol use. Females were 73% less likely to report risky alcohol use (AOR = 0.27;95% CI: 0.13–0.54). Prevalence of probable depression was 13.6% and female professionals were 5 times more likely to be classified as having probable depression (AOR = 4.86;95% CI: 1.08–21.90). The grouped prevalence of anxiety ranging from mild to severe was reported at 47.3%, female professionals were 3 times more likely to be classified as having anxiety disorder (AOR = 2.78;95% CI: 1.39–5.57). Furthermore, races other than African had higher rates of anxiety (AOR = 2.54; 95% CI: 1.00–6.42). The prevalence of suicide symptoms was 7.9% and that of covid dysfunctional anxiety 4.8%. Only 5% of participants were involved in an employee wellness program, with 60% expressing interest in joining one.

**Conclusion:**

Alcohol use (risky drinking) and mental disorders were common among healthcare professionals in West Rand District, Johannesburg, South Africa. There is overall poor awareness and use of support structures highlighting urgent need for interventions. Future studies could also explore in-depth the drivers of mental disorders and lack of utilization of the available service and strategies to deliver alcohol and mental disorder screening, brief intervention, and referral to treatment.

**Key messages**

- Risky alcohol use is common among male healthcare professionals.
- Mental disorders were common among female healthcare professionals.
- Awareness and utilization of support services was overall poor.
- There is a need for alcohol and mental disorders interventions.

## Introduction

Alcohol is a psychotropic substance able to affect mental processes such as effect and/or cognition and has dependence producing properties. Alcohol use is part of multiple social, religious and cultural practices due to its perceived ability to provide pleasure. Heavy alcohol use, however, is associated with greater risk for developing multiple medical conditions as well as mental disorders.^1^

During 2018, a global status report on alcohol and health estimated that 3 million alcohol related deaths occurred globally every year.^2^ Moreover during 2019, Statistica reported that South Africans consumed just under 10 L of alcohol per capita, making it the third most leading country in terms of alcohol consumption in Africa.^3^ In 2019, 1 in every 8 people, or 970 million people globally was found to have a mental disorder, depression and anxiety as commonest.^4^ These numbers are thought to have increased by 25% during the first year of the coronavirus disease 2019 (Covid-19) pandemic according to the World Health Organisation (WHO).^5^

There is a complex interplay of internal and external factors which eventually contribute to the use of alcohol and/or development of mental disorders. External factors include environment, family, religion, job status, and in many societies considered a cultural norm.^6^ Internal factors include genetics and psychological conditions such as other substance related disorders, antisocial personality disorder, mood disorders, and anxiety disorders.^6^

Healthcare professionals are highly vulnerable to developing risky alcohol use and mental disorders. They are exposed to long working hours, high workloads, concerns over medical errors and/or litigation, role conflict, emotional labour, and verbal and/or physical abuse.^7^ Additional stressors include lack of resources, personnel and critical facilities. Lastly exposure to dangerous pathogens such as Covid-19 can further exacerbate and strain healthcare professionals’ psychological state. Healthcare professionals are at the frontline when diagnosing, treating, and caring for patients with Covid-19. The ever-increasing numbers of cases depleted personal protective equipment, overwhelming workload, lack of clear guidelines and medication as well as fear of contracting or spreading the virus, have all contributed to the mental burden.^8^

During the coronavirus pandemic multiple studies conducted on healthcare professionals found alarmingly high rates of risky drinking. Problem drinking amongst healthcare professionals were reported in an American study (as high as 42.6%),^9^ a British study (7%)^10^ an Ethiopian study (40.2%),^11^ and a Kenyan study (43.9%).^12^ A rapid scoping review conducted in 2020 researched the mental health of healthcare professionals during the Covid-19 outbreak in South Africa and found prevalence of rates of depression (8.9%–50.4%) and anxiety (10.4%–44.6%).^13^

Both harmful alcohol use and abovementioned mental disorders can affect a healthcare professionals standard of care and overall safety to the patient.^14^ Risky drinking has been associated with reduced productivity and performance in the workplace due to cognitive impairment and ill health.^15^ Workplaces that encourage and support mental health are more likely to improve healthcare professionals’ quality of life, decrease absenteeism, increase productivity, and benefit from associated economic gains.^16^ Preventative mental health interventions or health promotion programmes are ways in which to reduce the risk of mental health problems within the workplace.

There is a lack of research on mental health in healthcare professionals working in South Africa particularly in primary care settings. Therefore, the aim of this study was to determine the prevalence and correlates of alcohol use and mental disorders including covid anxiety amongst healthcare professionals in West Rand District, Gauteng, South Africa. And in addition, assess the healthcare professionals’ awareness and utilization of support services.

## Methods

### Study design and setting

This study was a quantitative cross-sectional study. The research was conducted at 3 randomly selected Government Hospitals and Community Healthcare Centres (CHC) located in the West Rand District, Gauteng, South Africa. The 3 hospitals were Dr Yusuf Dadoo (District), Carletonville (District) and Leratong (Regional). The 3 Community HealthCare Centres were Bekkersdal West, Khutsong, and Mohlakeng. Recruitment was done by the investigator at each site. Participants were approached in their respective clinical work areas. The data were collected from June 2021 to March 2022.

### Study population

The inclusion criteria for participants included doctors (interns, community service, medical officers, registrars, and specialists), nurses (nursing professionals, nursing assistants, and professional nurses), clinical associates, and dentists. Since it was a survey on healthcare professionals, a positive response to participation was taken as an implied consent. Participants also had to be currently employed on the West Rand and aged 18 or older. Those who did not fulfil above criteria were excluded from the study.

### Sample size and sampling

According to the West Rand Health Council Area healthcare professionals totalled 2,747.^17^ Ideally, we would have wanted to include as many healthcare professionals as possible working in the West Rand District. However, having assumed that we could not access all healthcare professionals we calculated a sample size by using OpenEpi.^18^ Moreover, we assumed that 6% of healthcare professionals would have had an alcohol use disorder,^19^ using a population size of 2,747 healthcare workers with a precision of 3% and design effect of 2%. The minimal sample size was 443 healthcare professionals with a confidence interval of 95%.

### Data collection procedures

#### Recruitment of participants

Permission was obtained from the various heads of units to grant access to their facilities and to meet potential study participants. Recruitment was done by the investigator at each site. Study information was provided to all eligible participants who were then invited to partake in a paper-based self-administered questionnaire. Participants were provided with paper-based self-administered questionnaire that comprised of 7 sections with a total of 33 questions. The survey looked at sociodemographic data such as gender, age, marital status, religion, race, profession, work setting, and employment duration. Each collection site was provided a secure area to drop off surveys once completed. The investigator would then collect the surveys after a few days.

### Study variables

#### Outcomes (dependent variables)

Harmful alcohol use was measured by applying the 3-item question screening tool namely alcohol use disorder identification test (AUDIT-C). It is a shorter and modified version of the10 question AUDIT instrument. Studies have validated the use of the AUDIT-C for screening risky drinking and active abuse.^20^ The AUDIT-C measures alcohol misuse based on 3 questions about drinking habits. The scores are interpreted differently in males than in females. A score of 4 or more in males suggest alcohol misuse, while a score of 3 or more in females suggest the same.

Depression was measured using the patient health questionnaire-2 (PHQ-2). The full 9-item PHQ-9 questionnaire is a validated tool created by Kroenke et al.^21^ to assist in screening, diagnosing, and monitoring depression. The shorter version PHQ-2 is a 2-item questionnaire comprising of the first 2 questions from the PHQ-9,^22^ its main purpose is to screen for depression. A PHQ2 score can range from 0 to 6, if the score is equal to 3 or greater, major depression disorder is likely.^22^

The generalized anxiety disorder-7 (GAD-7) tool was used to screen for generalized anxiety disorder. It is a validated diagnostic tool for generalized anxiety.^23^ Each question is rated from zero to 3 with a maximum score of 21. A score of 10 and above indicates moderate symptom severity with possible clinically significant condition requiring further workup.^23,24^ A score of 15 and more is considered a severe symptom severity and warrants active treatment.^23,24^

The 3-item Patient Safety Scale-3 (PSS-3) is a validated, short and efficient tool used to screen for suicidality and ideation.^25^ The first 2 questions were based on thoughts experienced over the past 2 weeks and the last questions based on past experiences over a lifetime. Each question has 3 options to choose from: No, Yes, and refused to answer. A positive screening is found if ‘Yes’ was selected in question 2 or 3.^25^

The 5-item CAS questionnaire (CAS) was used to screen for anxiety related to Covid-19. It is a recently developed brief mental health screener for Covid-19-related anxiety, with 90% sensitivity and 85% specificity.^8^ Each question has 5 options to choose from, participants with a score of 9 and above are classified as having dysfunctional anxiety associated with the Covid-19 pandemic.^8^

The final part of the survey assessed the awareness of and utilization by healthcare professionals of organizations available for counselling on alcohol use and mental disorders. Multiple support services were provided, and healthcare professionals were asked to select the ones they had personally used in the past. Participants were asked if they were involved in any employee wellness program (EWP), interested in joining, and what types of services they would prefer.

#### Exposures (independent variables)

The study exposures included sociodemographic information such as job title, work environment, gender, race, religion, and marital status.

### Data management and analysis

The survey data were entered into a secure software platform called Research Electronic Data Capture (REDCap). Only the investigator and the supervisors had access to the data. Prior to analysis we recorded the scores used to assess the main outcomes (alcohol use and mental disorders).

We then computed descriptive statistics to summarize the sociodemographic information from the participants. Bivariate analysis (using chi-square or Fisher’s exact test) was used to determine the association between alcohol use, alcohol use disorders, mental disorders with the correlates. Furthermore, multivariable logistic regression was performed to determine the factors associated with alcohol use, alcohol use disorders, and mental disorders. From previous studies, age and sex were included in the multivariable models as a priori confounders of alcohol use, alcohol use disorder, and mental disorders.^12,26–29^ Other factors were entered in the multivariable models if they had a *P* value <0.20 in bivariate analysis (Supplementary Tables 1–4). We report the findings of multivariable analysis as adjusted odds ratios and corresponding 95% CI. In the final models, a *P* value of <0.05 was considered statistically significant.

## Results

A total of 500 surveys were disseminated with 330 surveys having been completed, this translates to 66% response rate.

### Sociodemographic characteristics of the study participants

Most of the participants were predominantly female (78.8%) with 48.2% of healthcare professionals aged between 35 and 64 years. The predominant religion was Christianity (87.3%) with mostly African healthcare professionals partaking (87.0%) in the study. Regarding healthcare profession 60.9% were nurses followed by doctors (33.0%), and 40.6% of the participants had been employed for a duration of more than 10 years. Additionally work setting varied amongst healthcare professionals with 36.1% working in wards and 35.8% working in the outpatient department and casualty (Table 1).

**Table 1.** General characteristics of healthcare professionals in West Rand District, Johannesburg, South Africa, 2021–2022.

Characteristic	Categories	*n*	%
Sex	Male	69	20.9
	Female	260	78.8
	Missing	1	0.3
	Total	330	100.0
Age (years)	23–34 years	119	36.1
	35–64 years	159	48.2
	Missing	52	15.8
	Total	330	100.0
Marital status	Single	125	37.9
	Married/with partner	180	54.5
	Widowed/divorced	24	7.3
	Missing	1	0.3
	Total	330	100.0
Religion	Christian	288	87.3
	Other	32	9.7
	Missing	10	3.0
	Total	330	100.0
			0.0
Race	Black/African	287	87.0
	Coloured	6	1.8
	Indian/Asian	12	3.6
	White	17	5.2
	Prefer not to disclose	4	1.2
	Missing	4	1.2
	Total	330	100.0
Race—binary	Black	287	87.0
	Others	39	11.8
	Missing	4	1.2
	Total	330	100.0
Profession	Medicine	109	33.0
	Nursing	201	60.9
	Other	18	5.5
	Missing	2	0.6
	Total	330	100.0
Work setting	Wards	119	36.1
	OPD and causality	118	35.8
	Other	73	22.1
	Missing	20	6.1
	Total	330	100.0
Employment duration	<4 years	92	27.9
	4–10 years	94	28.5
	>10 years	134	40.6
	Missing	10	3.0
	Total	330	100.0

OPD, outpatient department.

### Prevalence and factors associated with alcohol use disorder (risky drinking)

The overall prevalence of alcohol use disorder (AUD) amongst participants was (20.9%, *P* value <0.001). The AUD was highly prevalent amongst male healthcare professionals (42.0%, *P* value <0.001) when compared with females (15.4%, *P* value <0.001) (Table 2). In multivariable analysis (Table 3), only sex was associated with AUD and that females were less likely to report risky drinking (AOR = 0.27, 95% CI: 0.13–0.54).

**Table 2.** Prevalence of alcohol use disorder, depression, anxiety, suicide, COVID dysfunctional anxiety among healthcare professionals in West Rand District, Johannesburg, South Africa 2021–2022.

Characteristic	Categories	Sex						*P* value
		Male		Female		Total		
		*n*	%	*n*	%	*n*	%	
Alcohol use disorder (AUDIT-C)	No risky drinking	38	55.1	218	83.8	256	77.6	**<0.001**
	Risky drinking	29	42.0	40	15.4	69	20.9	
	Missing	2	2.9	2	0.8	5	1.5	
	Total	69	100.0	260	100.0	330	100.0	
Depression	No depression	64	92.8	210	80.8	274	83.0	**<0.001**
	Probable depression	2	2.9	43	16.5	45	13.6	
	Missing	3	4.3	7	2.7	11	3.3	
	Total	69	100.0	260	100.0	330	100.0	
Anxiety	No anxiety	44	63.8	120	46.2	164	49.7	**<0.001**
	Mild anxiety	19	27.5	82	31.5	101	30.6	
	Moderate anxiety	4	5.8	29	11.2	33	10.0	
	Severe anxiety	1	1.4	21	8.1	22	6.7	
	Missing	1	1.4	8	3.1	10	3.0	
	Total	69	100.0	260	100.0	330	100.0	
Anxiety (binary)	No anxiety disorder	44	63.8	120	46.2	164	49.7	**<0.001**
	Anxiety disorder	24	34.8	132	50.8	156	47.3	
	Missing	1	1.4	8	3.1	10	3.0	
	Total	69	100.0	260	100.0	330	100.0	
Suicide symptoms	No suicide symptoms	63	91.3	224	86.2	287	87.0	**<0.001**
	Suicide symptoms	2	2.9	24	9.2	26	7.9	
	Missing	4	5.8	12	4.6	17	5.2	
	Total	69	100.0	260	100.0	330	100.0	
COVID dysfunctional anxiety	No dysfunctional anxiety	68	98.6	240	92.3	308	93.3	**<0.001**
	Dysfunctional anxiety	0	0.0	16	6.2	16	4.8	
	Missing	1	1.4	4	1.5	6	1.8	
	Total	69	100	260	100.0	330	100.0	

The bolded *P*-value is statistically significant.

**Table 3.** Factors associated with alcohol use disorder among healthcare professionals in West Rand District, Johannesburg, South Africa, 2021–2022.

Characteristic	Categories	Adjusted OR	95% CI
Sex	Male	1	
	**Female**	**0.27** ^***^	**0.13–0.54**
Age (years)	23–34	1	
	35–64	0.52	0.26–1.02
Marital status	Single	1	
	Married/with partner	0.76	0.38–1.49
	Widowed/divorced	0.38	0.08–1.92
Religion	Christian	1	
	Other	0.87	0.23–3.22
Race	Black	1	
	Others	0.42	0.12–1.49
Probable depression	No	1	
	Yes	1.45	0.58–3.60
COVID dysfunctional anxiety	No	1	
	Yes	0.80	0.16–4.00

^***^ *P* < 0.001.

The bolded *P*-value is statistically significant.

### Prevalence and factors associated with depression

Regarding depression the overall prevalence for probable depression was 13.6% (*P* value <0.001), with females having markedly higher rates of probable depression (16.5%, *P* value <0.001) compared with males (2.9%, *P* value <0.001) (Table 2). In the multivariable analysis (Table 4) being female was significantly associated with increased odds of being classified as having probable depression (AOR = 4.86,95% CI: 1.08–21.90).

**Table 4.** Factors associated with probable depression among healthcare professionals in West Rand District, Johannesburg, South Africa, 2021–2022.

Characteristic	Categories	Adjusted OR	95% CI
Sex	Male	1	
	**Female**	**4.86** ^*^	**1.08–21.90**
Age (years)	23–34	1	
	35–64	1.34	0.60–3.03
Marital status	Single	1	
	Married/with partner	0.97	0.44–2.14
	Widowed/divorced	0.66	0.16–2.80
Religion	Christian	1	
	Other	0.65	0.17–2.41
Race	Black	1	
	Others	2.36	0.79–7.05
Alcohol use disorder	No	1	
	Yes	1.44	0.58–3.58
COVID dysfunctional anxiety	No	1	
	Yes	2.59	0.72–9.29

^*^ *P* < 0.05.

The bolded *P*-value is statistically significant.

### Prevalence and factors associated with anxiety disorder

The general prevalence of mild to severe anxiety was 47.3% (*n* = 156): mild anxiety 30.6% (*n* = 101); moderate anxiety 10% (*n* = 33); and severe anxiety 6.7% (*n* = 22). As presented in Table 2, females were found to have higher prevalence of anxiety than males (50.8% versus 34.8%, *P* value <0.001). In the multivariable analysis (Table 4) females were significantly associated with increased odds of anxiety (AOR = 2.78, 95% CI: 1.39–5.57). Furthermore, races other than African had higher percentages of anxiety (AOR = 2.54, 95% CI: 1.00–6.42) (Table 5).

**Table 5.** Factors associated with anxiety disorder among healthcare professionals in West Rand District, Johannesburg, South Africa, 2021–2022.

Characteristic	Categories	Adjusted OR	95% CI
Sex	Male	1	
	**Female**	**2.78** ^**^	**1.39–5.57**
Age (years)	23–34	1	
	35–64	0.90	0.51–1.60
Marital status	Single	1	
	Married/with partner	0.77	0.44–1.36
	Widowed/divorced	1.23	0.47–3.26
Religion	Christian	1	
	Other	1.20	0.43–3.35
Race	Black	1	
	Others	2.54^*^	1.00–6.42
Alcohol use disorder	No	1	
	Yes	1.60	0.83–3.08

^*^ *P* < 0.05.

^**^ *P* < 0.01.

The bolded *P*-value is statistically significant.

### Prevalence and factors associated with suicide symptoms

As presented in Table 2, the participants’ overall suicidal symptoms prevalence was 7.9% with females having higher prevalence than males (9.2% versus 2.9%, *P* value <0.001). In multivariable analysis (Table 6), being widowed/divorced was significantly associated with reporting suicidal symptoms (AOR = 6.88, 95% CI: 1.37–34.55).

**Table 6.** Factors associated with suicide symptoms among healthcare professionals in West Rand District, Johannesburg, South Africa, 2021–2022.

Characteristic	Categories	Adjusted OR	95% CI
Sex	Male	1	
	Female	7.88	0.98–63.19
Age (years)	23–34	1	
	35–64	0.36	0.13–1.03
Marital status	Single	1	
	Married/with partner	2.55	0.83–7.88
	Widowed/divorced	**6.88** *	**1.37–34.55**
Religion	Christian	1	
	Other	2.69	0.52–13.91
Race	Black	1	
	Others	0.16	0.02–1.14
Alcohol use disorder	No risky drinking	1	
	Risky drinking	1.32	0.42–4.13

*P < 0.05.

The bolded *P*-value is statistically significant.

### Prevalence of Covid-19 dysfunctional anxiety and correlates

The overall prevalence of Covid-19 dysfunctional anxiety was generally low amongst healthcare professionals (4.8%, *P* value <0.001) with no males having reported Covid-19 dysfunctional anxiety (Table 2). On bivariate analysis (Table 7), anxiety disorder (*P* < 0.001), probable depression (*P* = 0.031), and sex, being female (*P* = 0.034) were associated with Covid-19 dysfunctional anxiety. In multivariable analysis, none of the factors were associated with Covid-19 dysfunctional anxiety.

**Table 7.** Findings of the factors associated with COVID dysfunctional anxiety at bivariate analysis among healthcare professionals in West Rand District Johannesburg, South Africa, 2021–2022.

Characteristic	Categories	COVID dysfunctional anxiety					*P* value
		No		Yes		Total	
		*n*	%	*n*	%	*n*	
Sex	Male	68	100.0	0	0.0	68	**0.034**
	Female	240	93.8	16	6.3	256	
	Total	308	95.1	16	4.9	324	
Age (years)	23–34	112	94.1	7	5.9	119	0.795
	35–64	147	94.8	8	5.2	155	
	Total	259	94.5	15	5.5	274	
Marital status	Single	116	95.1	6	4.9	122	0.191
	Married/with partner	171	96.1	7	3.9	178	
	Widowed/divorced	21	87.5	3	12.5	24	
	Total	308	95.1	16	4.9	324	
Religion	Christian	269	94.7	15	5.3	284	0.183
	Other	32	100.0	0	0.0	32	
	Total	301	95.3	15	4.7	316	
Race	Black	267	94.7	15	5.3	282	0.459
	Others	38	97.4	1	2.6	39	
	Total	305	95.0	16	5.0	321	
Cadre	Medicine	106	97.2	3	2.8	109	0.422
	Nursing	183	93.8	12	6.2	195	
	Other	17	94.4	1	5.6	18	
	Total	306	95.0	16	5.0	322	
Working station	Wards	110	94.0	7	6.0	117	0.853
	OPD and causality	109	94.8	6	5.2	115	
	Other	70	95.9	3	4.1	73	
	Total	289	94.8	16	5.2	305	
Employment duration	<4 years	89	96.7	3	3.3	92	0.626
	4–10 years	88	94.6	5	5.4	93	
	>10 years	123	93.9	8	6.1	131	
	Total	300	94.9	16	5.1	316	
Alcohol use disorder	No	238	94.4	14	5.6	252	0.369
	Yes	67	97.1	2	2.9	69	
	Total	305	95.0	16	5.0	321	
Probable depression	No	259	96.3	10	3.7	269	**0.031**
	Yes	40	88.9	5	11.1	45	
	Total	299	95.2	15	4.8	314	
Suicide symptoms	No	270	95.4	13	4.6	283	0.126
	Yes	23	88.5	3	11.5	26	
	Total	293	94.8	16	5.2	309	
Anxiety disorder	No	161	99.4	1	0.6	162	**<0.001**
	Yes	139	90.3	15	9.7	154	
	Total	300	94.9	16	5.1	316	

The bolded *P*-value is statistically significant.

### Utilization and awareness of wellness services

Table 8 shows that there was low utilization of all the different helplines listed: Alcoholics Anonymous (AA) only 1.5% (*n* = 5); Alcoholics Anonymous for group of young people (Alteen) 1.5% *n* = 5; South African Depression and Anxiety Group (SADAG) 2.4% (*n* = 8); Depression and Mental Helpline (DMH) (*n* = 9); and lastly the Suicidal Crisis Helpline (SCH) only 0.6% (*n* = 2). The counselling service most used by healthcare professionals was psychology with 21.2% (*n* = 70). Most of the healthcare professionals 91.8% (*n* = 303) were not involved in EWP however 59.7% (*n* = 197) reported interest in joining one. Additionally, when asked what specific services were required, healthcare professionals reported as follows: 47% (*n* = 155) wellness events, 43.6% (*n* = 144) services, and 44.8% (*n* = 148) resources.

**Table 8.** Utilization of wellness services among healthcare professionals in West Rand District Johannesburg, South Africa 2021–2022.

Characteristic	Categories	Sex						*P* value
		Male		Female		Total		
		*n*	%	*n*	%	*n*	%	
Alcoholic Anonymous South Africa National Helpline (AA)	No	68	98.6	249	95.8	317	96.1	<0.001
	Yes	0	0.0	5	1.9	5	1.5	
		1	1.4	6	2.3	8	2.4	
	Total	69	100.0	260	100.0	330	100.0	
Alteen (group for young people with alcohol problems)	No	66	95.7	247	95.0	313	94.8	<0.001
	Yes	1	1.4	4	1.5	5	1.5	
		2	2.9	9	3.5	12	3.6	
	Total	69	100.0	260	100.0	330	100.0	
SADAG	No	66	95.7	244	93.8	310	93.9	<0.001
	Yes	1	1.4	7	2.7	8	2.4	
		2	2.9	9	3.5	12	3.6	
	Total	69	100.0	260	100.0	330	100.0	
Depression and Mental Health Help line	No	67	97.1	241	92.7	308	93.3	<0.001
	Yes	2	2.9	7	2.7	9	2.7	
		0	0.0	12	4.6	13	3.9	
	Total	69	100.0	260	100.0	330	100.0	
Suicide Crises line	No	66	95.7	244	93.8	310	93.9	0.001
	Yes	1	1.4	1	0.4	2	0.6	
		2	2.9	15	5.8	18	5.5	
	Total	69	100.0	260	100.0	330	100.0	
Psychologist	No	57	82.6	189	72.7	246	74.5	<0.001
	Yes	11	15.9	59	22.7	70	21.2	
		1	1.4	12	4.6	14	4.2	
	Total	69	100.0	260	100.0	330	100.0	
Are you currently involved in an employee wellness program?	No	63	91.3	240	92.3	303	91.8	<0.001
	Yes	4	5.8	12	4.6	16	4.8	
		2	2.9	8	3.1	11	3.3	
	Total	69	100.0	260	100.0	330	100.0	
Would you be interested in joining an employee wellness program if provided	No	31	44.9	78	30.0	109	33.0	0.001
	Yes	33	47.8	164	63.1	197	59.7	
		5	7.2	18	6.9	24	7.3	
	Total	69	100.0	260	100.0	330	100.0	
Wellness events (e.g. walking, nutrition, resilience programs)	No	3	4.3	9	3.5	12	3.6	0.270
	Yes	25	36.2	130	50.0	155	47.0	
		41	59.4	121	46.5	163	49.4	
	Total	69	100.0	260	100.0	330	100.0	
Services (e.g. screening, health coaching)	No	3	4.3	18	6.9	21	6.4	0.483
	Yes	26	37.7	118	45.4	144	43.6	
		40	58.0	124	47.7	165	50.0	
	Total	69	100.0	260	100.0	330	100.0	
Resources (e.g. online assessments, learning modules, training programs)	No	5	7.2	19	7.3	24	7.3	0.406
	Yes	25	36.2	123	47.3	148	44.8	
		39	56.5	118	45.4	158	47.9	
	Total	69	100.0	260	100.0	330	100.0	

## Discussion

These results reveal that healthcare professionals working in the West Rand have high levels of reported alcohol use disorder, anxiety, depression, and suicidality. Females were found to have much higher rates of anxiety, depression, suicidality, and Covid-19 anxiety. Males however had higher reported rates of alcohol use. Overall, there was poor utilization of support services in general with few healthcare professionals involved in EWP.

## Strengths/limitations

This is the first study conducted on healthcare professionals on the West Rand District, Gauteng province, South Africa to assess alcohol use disorder and a variety of mental health outcomes. This study provides important information on the prevalence and correlates of alcohol use disorder and mental health conditions among healthcare professionals. Our study was neither without limitations nor could the causation be inferred due to the nature of a cross-sectional study. Unfortunately, we were unable to meet our calculated sample size of 443 with only 330 completed surveys. Possible reasons for nonparticipation at recruitment included lack of time and being uninterested in research. This selection bias could lead to underestimation of the outcomes if those with alcohol use disorder (risky drinking) and mental disorders refrained to take part. Self-administered questionnaires were used for this study which relied on self-report measures, this may have caused recall bias. There can also be a tendency for healthcare professionals to under report harmful alcohol use/mental illness/suicidal ideation to be seen as safe and responsible due to the nature of their position thus social desirability bias may have occurred. To reduce the risk of response bias, surveys were anonymous, questions were short and clear and shorter screening tools for alcohol use disorder and depression.

## Interpretation

This cross-sectional study found that during the Covid-19 pandemic 20.9% of West rand healthcare professionals reported harmful alcohol use. Our findings vary from similar studies conducted in other countries during Covid-19 timeline. Our rate is much lower than healthcare professionals working in Kenya (43.9%) and United States of America (42.6%)^9,12^ and much higher than those in Italy (9.1%) and United Kingdom (7%).^10,26^ A possible reason for the discrepancy in alcohol prevalence rates could be due to the varied alcohol control policies across different countries and access to mental health services.

The high rate of harmful alcohol use found on the West Rand is a cause for concern especially in the male population. In our study being female was associated with decreased odds of harmful alcohol use, these findings are consistent with studies conducted in Kenya and Italy during the Covid-19 pandemic.^12,26^ These gender differences may be explained by strict cultural beliefs, values, and traditional gender roles that may prevent females from developing harmful substance abuse.^30^

Regarding depression 45 (13.6%) participants screened positive for probable depression. In contrast, Lai et al.^31^ conducted research at 34 hospitals in China and found much higher rates of depression (50%)_._ A systematic review titled ‘Global prevalence and burden of depressive and anxiety disorders in 204 countries and territories in 2020 due to the Covid-19 pandemic’ found higher rates of depression as compared with our population (24.3%).^32^ In our study females had higher chances of developing depression [AOR = 4.86; 95% CI: 1.08–21.90]. Naidoo et al. researched depression in healthcare professionals working in Kwa-Zulu Natal prior to the Covid-19 pandemic and similarly found an association between being female and having depression [OR 3.85, 95% CI: 1.21–12.22].^33^ A recent systematic review which researched the prevalence of major depressive disorder in the general population also reported females as more affected with depression than males.^34^ These differences may be attributed to prescribed gender roles, added stressors of balancing domestic and professional roles, empathetic tendencies, marginalization by male colleagues and the likelihood of social and economic consequences of the Covid-19 pandemic.^33,34^

Many respondents 47.3% (*n* = 101) in our study reported some form of anxiety. Robertson et al. conducted a systematic review of global literature surrounding mental health of healthcare professionals and found anxiety rates ranged anywhere from 10.4% to 44.6%.^13^ Differences in study population, screening tools and study sites may be causes of the wide range of prevalence rates.^13^ In our study being female was associated with increased odds of having anxiety [AOR = 2.78, 95% CI: 1.39–5.57]. A systematic review assessing mental health problems in healthcare professionals since Covid-19 found similar results to our study.^35^ They found that female workers, nurses, frontline healthcare workers, younger medical staff members and workers in higher infection areas experienced the highest level of psychological distress.^35^

Pre-Covid-19 studies showed consistently that both nurses and physicians were at a higher risk for suicide as compared with other employed people.^36,37^ Our study found higher rates of suicidal ideation (7.9%) as compared with the findings of 4.2% by Mortier et al. which was conducted at a large multicentre, in a prospective cohort study of Spanish healthcare workers (HCWs) active during the Covid-19 pandemic.^38^ The higher rate in our study could be due to South Africa as a developing country with limited resources, insufficient workforce, poor supervision/guidelines, and high job expectations. Rahman and Plummer found that females are at a higher risk of suicidal behaviour than males during the Covid-19 pandemic, this was true for our study whereby female healthcare professionals had higher suicidal prevalence than male health professionals (9.2% versus 2.9%, *P* value <0.001) and suicide symptoms were significantly higher among those divorced/widowed.^39^

Alarmingly there was overall poor utilization of helpline services besides psychology (21.2%, *n* = 70) amongst healthcare professionals considering the high rates of harmful alcohol use, depression, anxiety, and suicidality. A majority of healthcare professionals were not involved in any EWP 91.8% (*n* = 303) whereas oddly more than half 59.7% (*n* = 197) reported interest in joining one. When asked which services they would be interested in receiving from an EWP healthcare professionals reported health services, creating wellness events, and providing resources. Nikunlaakso et al. conducted a scoping review looking at interventions to reduce the risk of mental health problems in health workplaces.^40^ Their study revealed that there is a scarcity in the evidence that interventions reduce the risk of work-related mental health problems.^40^ However, psychological communication training and coaching, mental relaxation, muscle stressing, and emotional orientated care interventions were reported to have a positive effect and showed promising results.^40^

## Generalizability

Our sample was comprised mostly of nurses followed by doctors, a good representation as the latter makes up the largest group of healthcare providers in South Africa. The findings of this study could be extended in other settings in South Africa.

## Conclusions and recommendations

It is evident that harmful alcohol use, probable depression, anxiety, and suicidality are significant problems amongst healthcare professionals on the West Rand. Overall, there is poor utilization of support services but large interest in joining an EWP. There is a need to create and pilot, targeted interventions amongst healthcare professionals to improve awareness and utilization of the existing alcohol use and mental health services. Our recommendations include increasing awareness of the availability of alcohol use and mental health wellness programs/psychological services and to strengthen/create EWPs. Lastly there is also a need for integration of screening, brief interventions, and referral for treatment of alcohol use and mental disorders in EWPs. Future studies could also explore in-depth the drivers of mental disorders and lack of utilization of the available service and strategies to deliver alcohol and mental disorder screening, brief intervention, and referral to treatment.

## Supplementary Material

cmad094_suppl_Supplementary_Tables

cmad094_suppl_Supplementary_Checklist

## Data Availability

The datasets used in this study are available from the corresponding author upon reasonable request.
