# Supplementary material for: Prevalence and correlates of alcohol use, mental disorders, and awareness and utilization of support services among healthcare professionals in West Rand District, Gauteng, South Africa: a cross-sectional study
Source: Fam Pract. 2023 Sep 15;42(2):cmad094. doi: 10.1093/fampra/cmad094 (PMC11954498; doi:10.1093/fampra/cmad094)
Supplement: cmad094_suppl_Supplementary_Tables [file cmad094_suppl_supplementary_tables.docx]

**Supplementary Tables of results**

Supplementary Table 1: Findings of the factors associated with Alcohol Use Disorder at bivariate analysis among Health Care Providers in West Rand district, Johannesburg, South Africa, 2021- 2022

| Characteristic | Categories | Alcohol Use Disorder | | | | | P value |
| --- | --- | --- | --- | --- | --- | --- | --- |
|  |  | No | | Yes | | Total |  |
|  |  | n | % | n | % | n |  |
| Sex | Male | 38 | 56.7 | 29 | 43.3 | 67 | **<0.001** |
|  | Female | 218 | 84.5 | 40 | 15.5 | 258 |  |
|  | Total | 256 | 78.8 | 69 | 21.2 | 325 |  |
|  |  |  |  |  |  |  |  |
| Age | 23-34 | 82 | 69.5 | 36 | 30.5 | 118 | **0.004** |
|  | 35-64 | 132 | 84.1 | 25 | 15.9 | 157 |  |
|  | Total | 214 | 77.8 | 61 | 22.2 | 275 |  |
|  |  |  |  |  |  |  |  |
| Marital status | Single | 89 | 71.8 | 35 | 28.2 | 124 | **0.029** |
|  | Married/with partner | 145 | 81.9 | 32 | 18.1 | 177 |  |
|  | Widowed/divorced | 22 | 91.7 | 2 | 8.3 | 24 |  |
|  | Total | 256 | 78.8 | 69 | 21.2 | 325 |  |
|  |  |  |  |  |  |  |  |
| Religion | Christian | 224 | 78.6 | 61 | 21.4 | 285 | 0.493 |
|  | Other | 26 | 83.9 | 5 | 16.1 | 31 |  |
|  | Total | 250 | 79.1 | 66 | 20.9 | 316 |  |
|  |  |  |  |  |  |  |  |
| Race | Black | 220 | 77.5 | 64 | 22.5 | 284 | 0.088 |
|  | Others | 34 | 89.5 | 4 | 10.5 | 38 |  |
|  | Total | 254 | 78.9 | 68 | 21.1 | 322 |  |
|  |  |  |  |  |  |  |  |
| Cadre | Medicine | 80 | 75.5 | 26 | 24.5 | 106 | 0.604 |
|  | Nursing | 160 | 80.4 | 39 | 19.6 | 199 |  |
|  | Other | 14 | 77.8 | 4 | 22.2 | 18 |  |
|  | Total | 254 | 78.6 | 69 | 21.4 | 323 |  |
|  |  |  |  |  |  |  |  |
| Workstation | Wards | 95 | 80.5 | 23 | 19.5 | 118 | 0.341 |
|  | OPD and casualty | 93 | 80.2 | 23 | 19.8 | 116 |  |
|  | Other | 52 | 72.2 | 20 | 27.8 | 72 |  |
|  | Total | 240 | 78.4 | 66 | 21.6 | 306 |  |
|  |  |  |  |  |  |  |  |
| Employment duration | < 4 years | 68 | 75.6 | 22 | 24.4 | 90 | 0.698 |
|  | 4-10 years | 74 | 78.7 | 20 | 21.3 | 94 |  |
|  | >10 years | 106 | 80.3 | 26 | 19.7 | 132 |  |
|  | Total | 248 | 78.5 | 68 | 21.5 | 316 |  |
|  |  |  |  |  |  |  |  |
| Probable depression | No | 214 | 79.3 | 56 | 20.7 | 270 | 0.371 |
|  | Yes | 33 | 73.3 | 12 | 26.7 | 45 |  |
|  | Total | 247 | 78.4 | 68 | 21.6 | 315 |  |
|  |  |  |  |  |  |  |  |
| Anxiety disorder | No | 131 | 81.4 | 30 | 18.6 | 161 | 0.203 |
|  | Yes | 117 | 75.5 | 38 | 24.5 | 155 |  |
|  | Total | 248 | 78.5 | 68 | 21.5 | 316 |  |
|  |  |  |  |  |  |  |  |
| Suicide symptoms | No | 225 | 79.5 | 58 | 20.5 | 283 | 0.441 |
|  | Yes | 19 | 73.1 | 7 | 26.9 | 26 |  |
|  | Total | 244 | 79.0 | 65 | 21.0 | 309 |  |
|  |  |  |  |  |  |  |  |
| COVID dysfunctional anxiety | No | 238 | 78.0 | 67 | 22.0 | 305 | 0.369 |
|  | Yes | 14 | 87.5 | 2 | 12.5 | 16 |  |
|  | Total | 252 | 78.5 | 69 | 21.5 | 321 |  |

Supplementary Table 2: Findings of factors associated with probable depression at bivariate analysis among Health Care Providers in West Rand, Johannesburg, South Africa, 2021- 2022

| Characteristic | Categories | Probable Depression | | | | | P value |
| --- | --- | --- | --- | --- | --- | --- | --- |
|  |  | No | | Yes | | Total |  |
|  |  | n | % | n | % | n |  |
| Sex | Male | 64 | 97.0 | 2 | 3.0 | 66 | **0.004** |
|  | Female | 210 | 83.0 | 43 | 17.0 | 253 |  |
|  | Total | 274 | 85.9 | 45 | 14.1 | 319 |  |
|  |  |  |  |  |  |  |  |
| Age(years) | 23-34 | 103 | 86.6 | 16 | 13.4 | 119 | 0.809 |
|  | 35-64 | 130 | 85.5 | 22 | 14.5 | 152 |  |
|  | Total | 233 | 86.0 | 38 | 14.0 | 271 |  |
|  |  |  |  |  |  |  |  |
| Marital status | Single | 101 | 83.5 | 20 | 16.5 | 121 | 0.623 |
|  | Married/with partner | 153 | 87.4 | 22 | 12.6 | 175 |  |
|  | Widowed/divorced | 20 | 87.0 | 3 | 13.0 | 23 |  |
|  | Total | 274 | 85.9 | 45 | 14.1 | 319 |  |
|  |  |  |  |  |  |  |  |
| Religion | Christian | 239 | 86.0 | 39 | 14.0 | 278 | 0.813 |
|  | Other | 28 | 87.5 | 4 | 12.5 | 32 |  |
|  | Total | 267 | 86.1 | 43 | 13.9 | 310 |  |
|  |  |  |  |  |  |  |  |
| Race | Black | 242 | 87.1 | 36 | 12.9 | 278 | 0.090 |
|  | Others | 30 | 76.9 | 9 | 23.1 | 39 |  |
|  | Total | 272 | 85.8 | 45 | 14.2 | 317 |  |
|  |  |  |  |  |  |  |  |
| Cadre | Medicine | 94 | 87.9 | 13 | 12.1 | 107 | 0.745 |
|  | Nursing | 163 | 84.9 | 29 | 15.1 | 192 |  |
|  | Other | 15 | 83.3 | 3 | 16.7 | 18 |  |
|  | Total | 272 | 85.8 | 45 | 14.2 | 317 |  |
|  |  |  |  |  |  |  |  |
| Workstation | Wards | 101 | 86.3 | 16 | 13.7 | 117 | 0.831 |
|  | OPD and casualty | 99 | 86.1 | 16 | 13.9 | 115 |  |
|  | Other | 60 | 83.3 | 12 | 16.7 | 72 |  |
|  | Total | 260 | 85.5 | 44 | 14.5 | 304 |  |
|  |  |  |  |  |  |  |  |
| Employment duration | < 4 years | 79 | 87.8 | 11 | 12.2 | 90 | 0.552 |
|  | 4-10 years | 81 | 87.1 | 12 | 12.9 | 93 |  |
|  | >10 years | 108 | 83.1 | 22 | 16.9 | 130 |  |
|  | Total | 268 | 85.6 | 45 | 14.4 | 313 |  |
|  |  |  |  |  |  |  |  |
| Alcohol use disorder | No | 214 | 86.6 | 33 | 13.4 | 247 | 0.371 |
|  | Yes | 56 | 82.4 | 12 | 17.6 | 68 |  |
|  | Total | 270 | 85.7 | 45 | 14.3 | 315 |  |
|  |  |  |  |  |  |  |  |
| COVID Dysfunctional anxiety | No | 259 | 86.6 | 40 | 13.4 | 299 | **0.031** |
|  | Yes | 10 | 66.7 | 5 | 33.3 | 15 |  |
|  | Total | 269 | 85.7 | 45 | 14.3 | 314 |  |

Supplementary Table 3: Findings of factors associated with anxiety disorder at bivariate analysis among Health Care Providers in West Rand district, Johannesburg, South Africa, 2021-2022

| Characteristic | Categories | Anxiety Disorder | | | | | | | | P value | | |
| --- | --- | --- | --- | --- | --- | --- | --- | --- | --- | --- | --- | --- |
|  |  | No | | Yes | | | Total | |  | | |  |
|  |  | n | % | n | % | n | |  | | |  |  |
| Sex | Male | 44 | 64.7 | 24 | 35.3 | 68 | | **0.012** | | |  |  |
|  | Female | 120 | 47.6 | 132 | 52.4 | 252 | |  | | |  |  |
|  | Total | 164 | 51.2 | 156 | 48.8 | 320 | |  | | |  |  |
|  |  |  |  |  |  |  | |  | | |  |  |
| Age(years) | 23-34 | 56 | 47.5 | 62 | 52.5 | 118 | | 0.316 | | |  |  |
|  | 35-64 | 82 | 53.6 | 71 | 46.4 | 153 | |  | | |  |  |
|  | Total | 138 | 50.9 | 133 | 49.1 | 271 | |  | | |  |  |
|  |  |  |  |  |  |  | |  | | |  |  |
| Marital status | Single | 61 | 50.4 | 60 | 49.6 | 121 | | 0.803 | | |  |  |
|  | Married/with partner | 92 | 52.6 | 83 | 47.4 | 175 | |  | | |  |  |
|  | Widowed/divorced | 11 | 45.8 | 13 | 54.2 | 24 | |  | | |  |  |
|  | Total | 164 | 51.2 | 156 | 48.8 | 320 | |  | | |  |  |
|  |  |  |  |  |  |  | |  | | |  |  |
| Religion | Christian | 153 | 54.3 | 129 | 45.7 | 282 | | **0.029** | | |  |  |
|  | Other | 10 | 33.3 | 20 | 66.7 | 30 | |  | | |  |  |
|  | Total | 163 | 52.2 | 149 | 47.8 | 312 | |  | | |  |  |
|  |  |  |  |  |  |  | |  | | |  |  |
| Race | Black | 151 | 54.3 | 127 | 45.7 | 278 | | **0.006** | | |  |  |
|  | Others | 12 | 30.8 | 27 | 69.2 | 39 | |  | | |  |  |
|  | Total | 163 | 51.4 | 154 | 48.6 | 317 | |  | | |  |  |
|  |  |  |  |  |  |  | |  | | |  |  |
| Cadre | Medicine | 55 | 51.4 | 52 | 48.6 | 107 | | 0.710 | | |  |  |
|  | Nursing | 100 | 51.5 | 94 | 48.5 | 194 | |  | | |  |  |
|  | Other | 7 | 41.2 | 10 | 58.8 | 17 | |  | | |  |  |
|  | Total | 162 | 50.9 | 156 | 49.1 | 318 | |  | | |  |  |
|  |  |  |  |  |  |  | |  | | |  |  |
| Working station | Wards | 63 | 53.8 | 54 | 46.2 | 117 | | 0.399 | | |  |  |
|  | OPD and casualty | 57 | 49.6 | 58 | 50.4 | 115 | |  | | |  |  |
|  | Other | 31 | 43.7 | 40 | 56.3 | 71 | |  | | |  |  |
|  | Total | 151 | 49.8 | 152 | 50.2 | 303 | |  | | |  |  |
|  |  |  |  |  |  |  | |  | | |  |  |
| Employment duration | < 4 years | 42 | 46.7 | 48 | 53.3 | 90 | | 0.710 | | |  |  |
|  | 4-10 years | 46 | 50.5 | 45 | 49.5 | 91 | |  | | |  |  |
|  | >10 years | 68 | 52.3 | 62 | 47.7 | 130 | |  | | |  |  |
|  | Total | 156 | 50.2 | 155 | 49.8 | 311 | |  | | |  |  |
|  |  |  |  |  |  |  | |  | | |  |  |
| Alcohol use disorder | No | 131 | 52.8 | 117 | 47.2 | 248 | | 0.203 | | |  |  |
|  | Yes | 30 | 44.1 | 38 | 55.9 | 68 | |  | | |  |  |
|  | Total | 161 | 50.9 | 155 | 49.1 | 316 | |  | | |  |  |
|  |  |  |  |  |  |  | |  | | |  |  |
| COVID dysfunctional anxiety | No | 161 | 53.7 | 139 | 46.3 | 300 | | **<0.001** | | |  |  |
|  | Yes | 1 | 6.3 | 15 | 93.8 | 16 | |  | | |  |  |
|  | Total | 162 | 51.3 | 154 | 48.7 | 316 | |  | | |  |  |

Supplementary Table 4: Findings of factors associated with suicide symptoms at bivariate analysis among Health Care Providers in West Rand district, Johannesburg, South Africa 2021-2022

| Characteristic | Categories | Suicide symptoms | | | | | P value |
| --- | --- | --- | --- | --- | --- | --- | --- |
|  |  | No | | Yes | | Total |  |
|  |  | n | % | n | % | n |  |
| Sex | Male | 63 | 96.9 | 2 | 3.1 | 65 | 0.086 |
|  | Female | 224 | 90.3 | 24 | 9.7 | 248 |  |
|  | Total | 287 | 91.7 | 26 | 8.3 | 313 |  |
|  |  |  |  |  |  |  |  |
| Age (Years) | 23-34 | 102 | 90.3 | 11 | 9.7 | 113 | 0.610 |
|  | 35-64 | 139 | 92.1 | 12 | 7.9 | 151 |  |
|  | Total | 241 | 91.3 | 23 | 8.7 | 264 |  |
|  |  |  |  |  |  |  |  |
| Marital status | Single | 110 | 94.0 | 7 | 6.0 | 117 | 0.187 |
|  | Married/with partner | 158 | 91.3 | 15 | 8.7 | 173 |  |
|  | Widowed/divorced | 19 | 82.6 | 4 | 17.4 | 23 |  |
|  | Total | 287 | 91.7 | 26 | 8.3 | 313 |  |
|  |  |  |  |  |  |  |  |
| Religion | Christian | 252 | 92.0 | 22 | 8.0 | 274 | 0.751 |
|  | Other | 28 | 90.3 | 3 | 9.7 | 31 |  |
|  | Total | 280 | 91.8 | 25 | 8.2 | 305 |  |
|  |  |  |  |  |  |  |  |
| Race | Black | 250 | 91.6 | 23 | 8.4 | 273 | 0.948 |
|  | Others | 34 | 91.9 | 3 | 8.1 | 37 |  |
|  | Total | 284 | 91.6 | 26 | 8.4 | 310 |  |
|  |  |  |  |  |  |  |  |
| Cadre | Medicine | 96 | 91.4 | 9 | 8.6 | 105 | 0.460 |
|  | Nursing | 173 | 91.1 | 17 | 8.9 | 190 |  |
|  | Other | 16 | 100.0 | 0 | 0.0 | 16 |  |
|  | Total | 285 | 91.6 | 26 | 8.4 | 311 |  |
|  |  |  |  |  |  |  |  |
| Working station | Wards | 105 | 92.9 | 8 | 7.1 | 113 | 0.409 |
|  | OPD and casualty | 101 | 91.8 | 9 | 8.2 | 110 |  |
|  | Other | 62 | 87.3 | 9 | 12.7 | 71 |  |
|  | Total | 268 | 91.2 | 26 | 8.8 | 294 |  |
|  |  |  |  |  |  |  |  |
| Employment duration | < 4 years | 78 | 91.8 | 7 | 8.2 | 85 | 0.901 |
|  | 4-10 years | 84 | 92.3 | 7 | 7.7 | 91 |  |
|  | >10 years | 116 | 90.6 | 12 | 9.4 | 128 |  |
|  | Total | 278 | 91.4 | 26 | 8.6 | 304 |  |
|  |  |  |  |  |  |  |  |
| Alcohol use disorder | No | 225 | 92.2 | 19 | 7.8 | 244 | 0.441 |
|  | Yes | 58 | 89.2 | 7 | 10.8 | 65 |  |
|  | Total | 283 | 91.6 | 26 | 8.4 | 309 |  |
|  |  |  |  |  |  |  |  |
| COVID dysfunctional anxiety | No | 270 | 92.2 | 23 | 7.8 | 293 | 0.126 |
|  | Yes | 13 | 81.3 | 3 | 18.8 | 16 |  |
|  | Total | 283 | 91.6 | 26 | 8.4 | 309 |  |
